# Monitoring and Assessment of Water Level Fluctuations of the Lake Urmia and Its Environmental Consequences Using Multitemporal Landsat 7 ETM^+^ Images

**DOI:** 10.3390/ijerph17124210

**Published:** 2020-06-12

**Authors:** Viet-Ha Nhu, Ayub Mohammadi, Himan Shahabi, Ataollah Shirzadi, Nadhir Al-Ansari, Baharin Bin Ahmad, Wei Chen, Masood Khodadadi, Mehdi Ahmadi, Khabat Khosravi, Abolfazl Jaafari, Hoang Nguyen

**Affiliations:** 1 Geographic Information Science Research Group, Ton Duc Thang University, Ho Chi Minh City 700000, Vietnam; nhuvietha@tdtu.edu.vn; 2 Faculty of Environment and Labour Safety, Ton Duc Thang University, Ho Chi Minh City 700000, Vietnam; 3 Department of Remote Sensing and GIS, University of Tabriz, Tabriz 51666-16471, Iran; mohammadi.ayub@tabrizu.ac.ir; 4 Department of Geomorphology, Faculty of Natural Resources, University of Kurdistan, Sanandaj 66177-15175, Iran; h.shahabi@uok.ac.ir; 5 Board Member of Department of Zrebar Lake Environmental Research, Kurdistan Studies Institute, University of Kurdistan, Sanandaj 66177-15175, Iran; 6 Department of Rangeland and Watershed Management, Faculty of Natural Resources, University of Kurdistan, Sanandaj 66177-15175, Iran; a.shirzadi@uok.ac.ir; 7 Department of Civil, Environmental and Natural Resources Engineering, Lulea University of Technology, 971 87 Lulea, Sweden; 8 Department of Geoinformation, Faculty of Built Environment and Surveying, Universiti Teknologi Malaysia (UTM), Johor Bahru 81310, Malaysia; baharinahmad@utm.my; 9 College of Geology & Environment, Xi’an University of Science and Technology, Xi’an 710054, China; chenwei0930@xust.edu.cn; 10 Key Laboratory of Coal Resources Exploration and Comprehensive Utilization, Ministry of Natural Resources, Xi’an 710021, China; 11 School of Business and Creative Industries, University of the West of Scotland, Paisley PA1 2BE, UK; masood.khodadadi@uws.ac.uk; 12 Department of Geomorphology, Faculty of Planning and Environmental Sciences, University of Tabriz, Tabriz 51666-16471, Iran; mehdi.ahmadi2009@gmail.com; 13 School of Engineering, University of Guelph, Guelph, ON N1G 2W1, Canada; kkhosrav@uoguelph.ca; 14 Research Institute of Forests and Rangelands, Agricultural Research, Education, and Extension Organization (AREEO), Tehran P.O. Box 64414-356, Iran; jaafari@rifr-ac.ir; 15 Institute of Research and Development, Duy Tan University, Da Nang 550000, Vietnam

**Keywords:** Lake Urmia, Iran, water level fluctuation, environmental consequences, remote sensing, GIS

## Abstract

The declining water level in Lake Urmia has become a significant issue for Iranian policy and decision makers. This lake has been experiencing an abrupt decrease in water level and is at real risk of becoming a complete saline land. Because of its position, assessment of changes in the Lake Urmia is essential. This study aims to evaluate changes in the water level of Lake Urmia using the space-borne remote sensing and GIS techniques. Therefore, multispectral Landsat 7 ETM^+^ images for the years 2000, 2010, and 2017 were acquired. In addition, precipitation and temperature data for 31 years between 1986 and 2017 were collected for further analysis. Results indicate that the increased temperature (by 19%), decreased rainfall of about 62%, and excessive damming in the Urmia Basin along with mismanagement of water resources are the key factors in the declining water level of Lake Urmia. Furthermore, the current research predicts the potential environmental crisis as the result of the lake shrinking and suggests a few possible alternatives. The insights provided by this study can be beneficial for environmentalists and related organizations working on this and similar topics.

## 1. Introduction

Reservoirs, wetlands, and lakes make water available to industrial, domestic, irrigation, and environmental areas [1,2]. The water level is the key component of these natural phenomena, which are mostly determined through their coastlines, which are, in turn, recognized as the borderline between the land and the water body. Forecasting lake water level at any scale is an essential concern in water resource planning and catchment management, management of hydropower plants, commercial navigation, and domestic, agricultural, and industrial activities in many countries [3]. Variables including incoming and outgoing water discharges, precipitation rate within the basin, groundwater harvesting, and evaporation are among the most determining factors affecting lake water level fluctuations [4]. However, applying these factors for such a purpose may result in low prediction accuracy since the prediction of lake water level is greatly controlled by complex hydrometeorological and anthropogenic factors [5,6,7,8] that cannot be easily and quickly assessed [6]. Accurate and regular monitoring of lakes and reservoirs water level variations is crucial for fair and equitable water allocation to different sectors, ecosystem services, and for better understanding of climate change impacts [9].

Lake Urmia is the world’s second largest hypersaline lake and one of the most important water bodies in Iran as well. It significantly affects the environment and economy of the northwestern parts of the country [10]. Even though the lake has shown a positive trend in its water level from 1966 to 1995, it has experienced significant decrease in water-level over the recent years, roughly 6 m between June 1995 and May 2009. Climate change, excessive dam construction, agricultural usage, and mismanagement of water resources resulted in a decreased water level of the Lake Urmia since 1995. It has led to a remarkable reduction of the lake’s surface area, high salinity of more than 340 mg/L [11], and the emergence of salty lands, which has caused a variety of ecological and environmental problems [12,13,14], such as increased nutrient concentrations and phytoplankton biomass favoring cyanobacterial blooms [15], loss of aquatic habitats [16], high concentration of sulfate and the trace elements like arsenic, Zn, and Pb [17], and destruction of lake’s ecosystem function and integrity [18]. These problems have attracted researchers’ attention to focus on the lake’s environmental health more delicately.

Because of its special biodiversity, Lake Urmia was declared a National Park, Ramsar Site since 1971, and a Biosphere Reserve by UNESCO since 1976 [19]. Due to the importance of this lake to the environment, health and, wealth of people residing in the northwest of Iran, identifying the fluctuation behavior of the lake’s water level is significant for planning and designing hydraulic attributes [4]. Most of the previous researches on the water level fluctuation either at global or local scales have concentrated on the significance of declining water body as well as decreasing river discharge into lakes (e.g., [2,3,9,13,20,21,22,23,24,25,26,27]), while taking a holistic approach by focusing on all aspects of water level declining has been markedly given less attention. Measuring and modeling are two valuable approaches for mapping water level fluctuations of any water body. Even though measuring is always preferable, it is costly, time consuming, and needs extensive labor works [28]. Hence modeling is suggested as an attractive alternative for purposes of this study. Furthermore, according to Tien Bui et al. [29], there is no single best model for different environmental problems, and thus, the development and assessment of new models should be continued.

To predict water level variations in lakes, numerous models have been used that include neuro-fuzzy, neural networks, and genetic programming models [30,31,32]. Both measuring and modeling inherit intrinsic drawbacks so that their combination may improve our insight into the lake’s structural function and integrity due to water level fluctuation.

The main objective of the present research is to evaluate temporal water level variations of Lake Urmia in 2017 compared to 2000 and 2010. The study used the most appropriate tools for drastic changes in water level suggested by Berrocoso et al. [33] such as multispectral Landsat 7 images of ETM^+^, 3D, GIS, and a statistical model which applies a maximum likelihood approach. Other objectives of the current work are to point out the effects of the Lake Urmia shrinking on the environment and suggesting different management alternatives to cope with this problem.

## 2. Study Area

**Figure 1:**
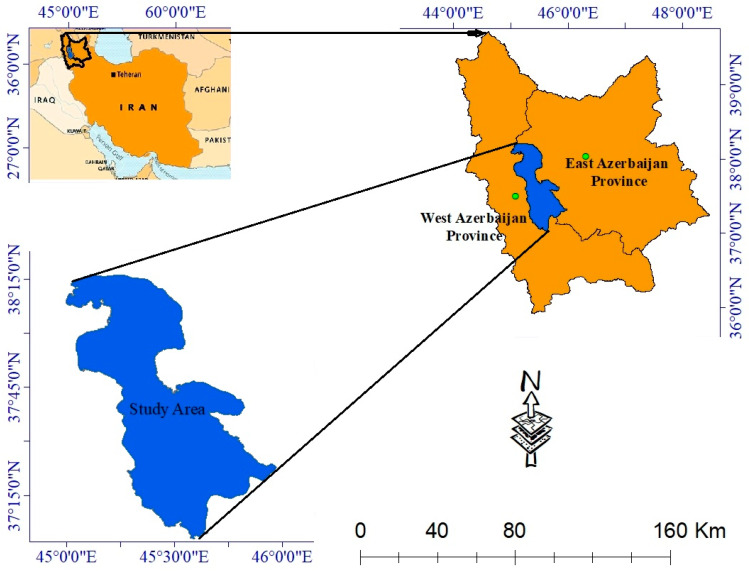
Geographical location of the Lake Urmia.

The Urmia Lake is situated in the northwest of Iran, approximately between 45°00′ and 46°00′ East longitudes and 37°00′ to 38°16′ North latitudes. We extracted the study area based on the water body of the year 2000 (Figure 1). The lake’s water is very saline such that only some species of crustaceans (Artemia), diatoms, and phytoplanktons can cope with it. However, the Lake Urmia National Park, whose surface area varies between 4750 and 6100 km^2^, is one of the nine biosphere reserves in Iran, where 212 bird species, 41 reptiles’ species, 27 species of mammal, 26 species of fish, and 7 amphibian’s species consist its wildlife [25,34]. Lake Urmia is regarded as a shallow lake with a maximum depth of 16 m [35], 1275.6 m above sea level [36], and an area of 5067.142 km^2^. It is the biggest lake in the Middle East. According to Emdadi et al. [37], the average annual precipitation and average annual temperature of the lake are about 341 mm and 11.2 °C, respectively. Moreover, the lake is the second largest hypersaline lake worldwide with total dissolved salts of 200 g/L in comparison with the normal salinity of approximately 35 g/L [37,38,39,40].

## 3. Materials and Methods

Landsat 7 was launched and placed in orbit successfully on 15 April 1999, 18:32:00 UTC (Landsat 7 handbook). Since our research was from 2000 to late 2017, the Landsat 7 ETM^+^ imagery was selected as suitable imagery. However, the Scan Line Corrector (SLC) of the Landsat 7 program from the year 2003 onwards has been turned off and resulted in some spatial gaps in the imageries. In this study, a total number of nine predominantly Landsat 7 ETM^+^ images for the years 2000, 2010, and 2017 were obtained from the US Geological Survey (USGS) website. Table 1 details the characteristics of the three images for three years of 2000, 2010, and 2017 and date of acquired images, respectively.

As shown in Table 1, only for path 168, row 34 (year of 18/10/2000), the temporal gap is about 2 months. Both availability and spatial gaps are reasons for this matter. Availability of data is a quite clear reason, but regarding the spatial gap, from 2003 onwards, the Scan Line Corrector (SLC) of Landsat 7 mission has been facing a severe problem which resulted in spatial gaps. However, in some images, these spatial gaps are too much confusing and results in noises, which will affect extracting Region of Interests (ROIs) and then classification. 

**Figure 2:**
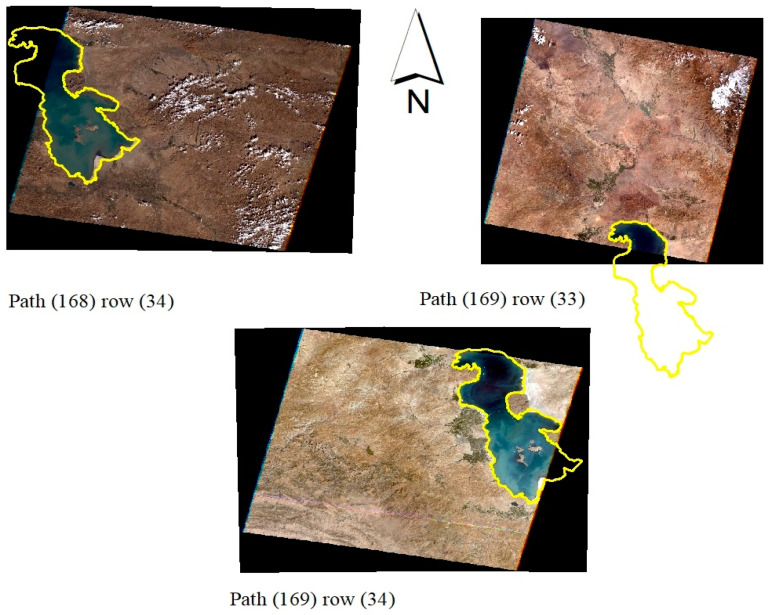
Geographical position of the satellite imageries used for the study area.

It should be noted that the vast extent of the study area is covered by the images of path 169 row 34 (22/08/2000) while the smallest part of the study area was clipped from the available path 168 row 34 (18/10/2000) imagery (Figure 2).

### 3.1. Dam, River, Precipitation, and Temperature Data 

Latitude and longitude of the dams were gathered from the Iran Water Resources Management Company (IWRMC) and mapped. In this study, the mean annual temperature and total annual precipitation for a 31-year period (1986–2017) were acquired from the Iran Meteorological Organization (IRIMO) to evaluate the water level of Lake Urmia. Temperature and precipitation data were analyzed to examine the trend of climatological factors of the lake and their impact on decreasing water levels from the year 2000 onwards. The x-axis (raw data) represented the names of rivers of Urmia Basin and the y-axis represented percentage of water supplied that is acquired from IWRMC and mapped using the Microsoft Excel 2010. 

**Figure 3:**
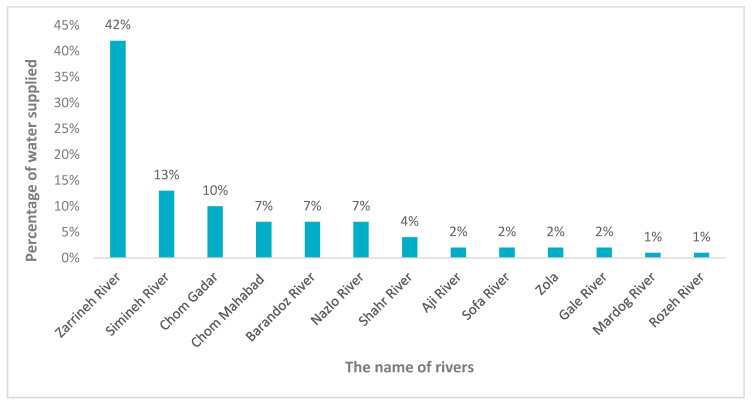
Main rivers’ discharge into the Lake Urmia.

About 13 major rivers drainage surface streams in the Urmia Basin, contributing ~6000 million cubic meters of water annually to the lake. [37]. The Simineh and Zarrineh Rivers are the main perennial rivers, originating from the Zagros Mountains of the Kurdistan provinces in the southeast part of the Lake Basin, in the Miandoab alluvial plain. About 50% of the water reaching Lake Urmia is from these two rivers [38]. The Zarrineh River is the largest river of the basin, supplying 42% of the lake water, while 13% of the water is supplied by the Simineh River that is the second largest supplier of the lake. However, damming on these rivers and their tributaries from the year 2000 onwards, which are the most important suppliers of Lake Urmia, has resulted in a decreased percentage of water supplies of the rives and their tributaries (Figure 3).

Furthermore, the water amount that pours into the Lake Urmia by the aforementioned rivers depends greatly on the season. For instance, during the spring season, the Talkheh and Simineh Rivers each can release about 2000 cubic feet (57 m^3^) to the lake per second, while this rate unbelievably will decrease to only 60–130 cubic feet (3.7–1.7 m^3^) per second during the mid-summers. This is the cause of the lake water-level fluctuations by 2–3 feet (0.6–0.9 m). Apart from these seasonal variations, there is also a longer period of fluctuations (lasting from 12–20 years) which led to the water-level fluctuation of 6–9 feet (1.8–2.7 m) (www.britannica.com).

### 3.2. Methodology 

Preprocessing of the satellite imageries before evaluating changes is a crucial step for increasing the accuracy of results [41,42,43]. Data preprocessing was carried out to provide a radiometrically, atmospherically, spectrally, and geometrically corrected imageries. Since, based on the location of the study area, we have acquired three images that must be mosaicked. However, the imageries using the panchromatic band were pan sharpened to 15 m. After the classification process, some unwanted pixels found within different land covers were removed using the majority filter available in postclassification toolbox of ENVI software (Broomfield, Colorado, CO, USA).

Landsat 7 satellite imageries were used for generating final maps, while from the year 2003 onwards, the SLC of Landsat 7 faced a problem and resulted in spatial gaps in imageries [44]. Without the SLC operation, the ETM^+^ line of sight causes a zig-zag pattern along the ground track of the satellite. Therefore, it creates some stripes on images so they must be gap-filled through ENVI Software (ENVI has a module to correct these gaps) or any other related software. There are two accepted ways for filling these gaps, one of them is using another imagery of the same scene but different time (in which the missing lines can be retrieved), while another method is to use the upper and the lower parts of the same imagery (because all imageries from 2003 onwards have the same problem, this method used to fill the spatial gaps of the imageries for the years 2010 and 2017 in this study).

**Figure 4:**
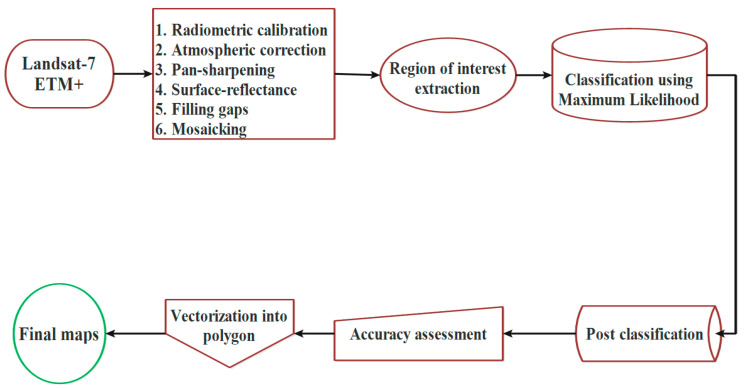
Flowchart of the research.

On the other side, after filling the gaps, the images are not perfect yet and the gaps cannot be completely removed, here Region of Interests (ROIs) extraction and classification method are important. After extracting ROIs, different algorithms of Maximum Likelihood (ML), Minimum Distance (MD), Spectral Angle Mapper (SAM), Support Vector Machine (SVM), and Artificial Neural Network (ANN) were used. Compared to the Google Earth images of the same date and also less unwanted pixels in the final map, the Maximum Likelihood model was selected as the best one among all for classification in this study. Before the validation step, postclassification processes were applied to the maps. Finally, using the ArcGIS software, the maps were vectorized into polygons as final maps (Figure 4).

#### Maximum Likelihood Method

According to Richards and Richards [45], Maximum Likelihood (ML) decides the statistical values for each class in bands with a normal distribution, therefore it measures the probability in which a given pixel allocates to a special class and each pixel will be assigned to a class, which has the highest probability. The pixel remains unclassified if the probability is smaller than a threshold. Maximum Likelihood classification was calculated by the following equation:

(1) gi(x)−1n p(∞i)−121n|∑i|−12(x−mi)∑i−1 (x−mi)

where $g_{i}$ is the number of classes, *x* is the number of satellite imagery sands, *p*($\infty_{i}$) is probability in which class $\infty_{i}$ occurs in the image and is expected the same for all classes, $\left|\sum_{i}\right|$ is determinant of the covariance matrix of the data in class $\infty_{i}$, $\sum_{i}-1$ is its inverse matrix, and *m_i_* is mean vector.

### 3.3. Accuracy Assessment

Confusion matrix, as an accuracy assessment method, was used to validate the statistical classification of the current research. Different accuracy assessment methods are available, such as overall accuracy (OA) and kappa. OA is one of the simplest and popular accuracy methods. It is calculated by dividing the total correct pixels (i.e., the sum of the major diagonal) by the total number of error matrix’s pixels [46]. Accuracy of the individual categories can be calculated in a similar procedure as well. Story and Congalton [47] developed (1) “producer’s accuracy” to demonstrate the probability of a reference pixel that has been correctly classified and (2) “user’s accuracy” to represent the probability in which a pixel classified on the map signifies category on the ground.

The overall accuracy (OA) is achieved by calculating the number of corrected classified pixels (CCPs) and then dividing by the total number of pixels [48].

(2) $$OA=\frac{1}{N}\sum p_{i}$$

where *OA* is total accuracy, *N* is the total number of test pixels, and $\sum p_{i}$ is the total pixels that are correctly classified.

Kappa coefficient (K) as one of the popular accuracy assessment methods, which measures inter-rater agreement for qualitative (categorical) items. However, it is renowned as a more robust method in comparison with the simple percent calculation. Because the possibility of the agreement that occurs by chance will be taken into account by the κ method. This coefficient ranges from −1 (nonreliable) to +1 (reliable) [49]. Furthermore, the output values of ≤0, 0–0.2, 0.2–0.4, 0.4–0.6, 0.6–0.8, and 0.8–1 indicate poor, slight, fair, moderate, substantial, and almost perfect agreement between the estimation (the model outputs) and the observation (the reality), respectively [50]. Provided that the two maps have the categories with a similar data, the values have a real meaning [51]. The kappa coefficient is achieved by the following formula:

(3) K=(OA−1/q) (1−1/q)

where *K* is kappa coefficient and *q* is unclassified pixels.

Google Earth imageries are quite good source of information for accuracy assessment of land covers, in particular, water bodies. However, for each class, 30 points were randomly extracted and used as Ground Control Points (GCPs) for the same dates and time as satellite data. 

## 4. Results and Analysis

### 4.1. Climate Change (Temperature and Precipitation)

**Figure 5:**
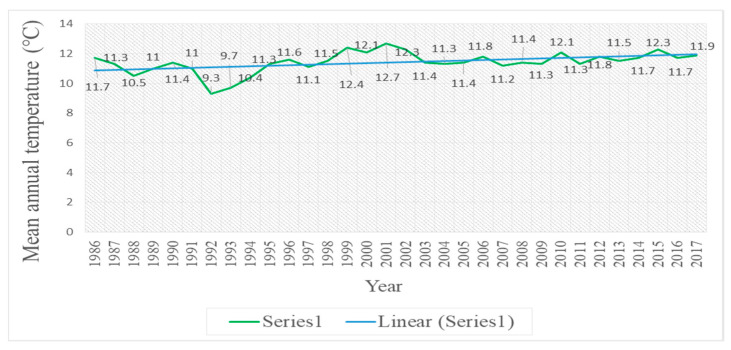
Temperature values of the Lake Urmia from 1986–2017.

Results show that the increased temperature and decreased rainfall, as well as excessive damming in the Urmia Basin are key factors in declining of the water level. Based on the analysis of the temperature data, the gradually increased temperature from 1986–2017 could be considered as one of the main reasons for the declining of water body of the lake. On the other hand, the average annual temperature in the northwest of Iran will reach about 23 °C in the next few decades [52], which itself leads to a climate change from semidesert to desert. It is noted that, average annual temperature in the northwest of Iran will decrease by 3%. Investigation over the mean annual temperature values demonstrates that the average temperature was nearly 9.7 °C until 1996 in the study area [37]. However, the temperature began to increase from 1996 onwards. The raising rate from 1996 to 2001 is estimated to be nearly 12.7% [37] and from 1986 to 2017 was nearly 19% (Figure 5). 

**Figure 6:**
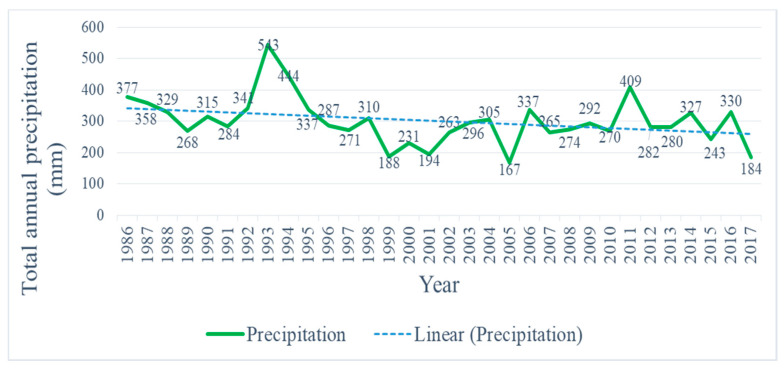
Precipitation values of Urmia Lake from 1986 to 2017.

Furthermore, the analysis of the precipitation data indicates that another major testimony for water subsidence in Lake Urmia is the rainfall reduction from 1986–2017. Figure 5 clearly illustrates that the continuous decline in precipitation values is another noticeable factor in shrinking out of the water level of Lake Urmia. A significant decline was seen in precipitation from 1986–2017, where it was 377 mm in the year 1986 and only 184 mm by 2017. The total annual precipitation from 1986–2017 has a decreased value of about 62% (Figure 6).

**Figure 7:**
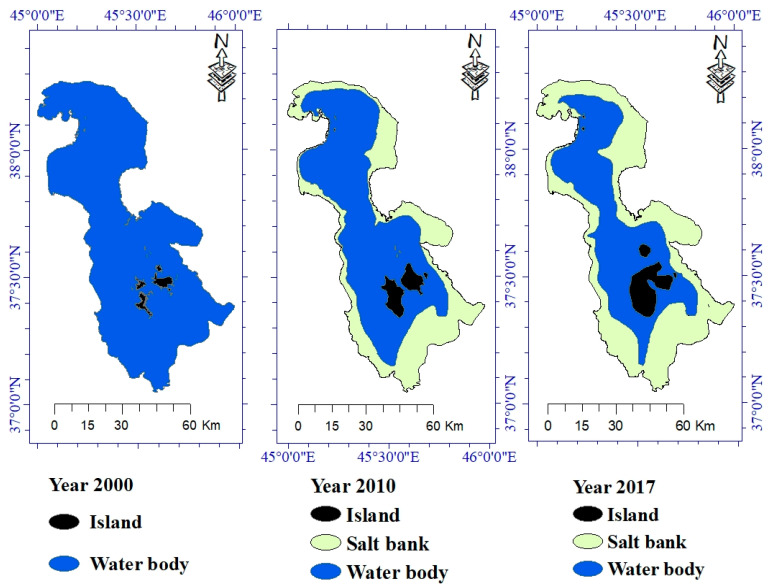
The final change maps of the study area extracted by the Landsat 7 ETM^+^ imageries for the years 2000, 2010, and 2017.

One of the main goals of this study was to map water level, island, and salt bank in 2000, 2010, and 2017 and changes occurred through Landsat 7 ETM^+^ imageries. As can be seen from the produced maps (Figure 7), the water body is less than the salt area in 2017. This declining trend may continue in the near future such that Lake Urmia turns into a salty land. 

### 4.2. Extracting Water Level in the Years 2000, 2010, and 2017

**Figure 8:**
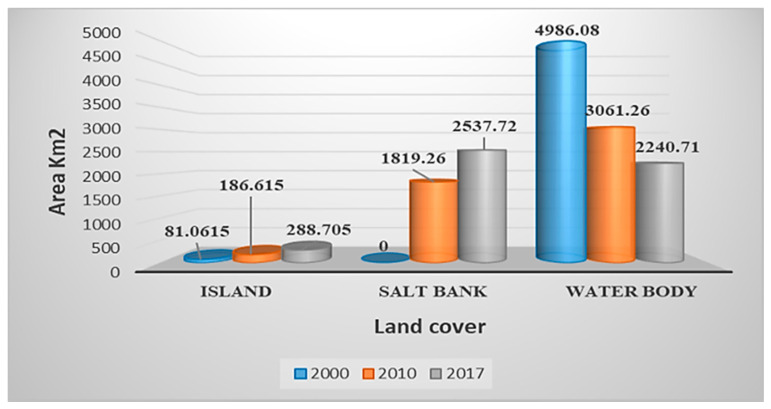
Changes in different land cover area (km^2^).

Lake Urmia water level has significantly declined which, in turn, has increased its salinity and affected water quality. Since we extracted the study area based on the water body of the lake, we do not have a value for the salt bank in 2000. In 2000, the island area was only 81.0615 km^2^, while because of a reduction in water level; it has dramatically been increased to 288.705 km^2^ by 2017. However, the water body has seen considerable reductions in its wet surface, from 4986.08 km^2^ in the year 2000 to 3061.26 km^2^ and 2240.71 km^2^ in 2010 and 2017, respectively. Water body decline will lead to an expanding salt bank. Table 2, Table 3 and Table 4 and Figure 8 clearly show the changes in land area from 2000 to 2017.

### 4.3. Validation

Validity and reliability make any research countable and referable to other scholars. In this study, the overall accuracy and kappa coefficient using the confusion matrix were measured for all three maps. Kappa coefficient for the maps of the years 2000, 2010, and 2017 was 0.9634, 0.9574, and 0.9384, respectively. Further, overall accuracy was more than 96% for all three maps (Table 5), proving that the work is well validated.

## 5. Discussion

### 5.1. Causes of the Lake Shrinking

Unplanned dam construction due to inappropriate management, drought occurrences (i.e., increased temperature and decreased precipitation), excessive groundwater exploitation by farmers, and illegal and irregular development of wells are among the main reasons for the decrease of water level in Lake Urmia. The lake’s surface area has been estimated to be as large as 6100 km^2^ in 1997. However, since 1995, it has generally been declining and in August 2011, it was estimated, based on satellite data, to be only 2366 km^2^ [10]. Furthermore, the water level of Lake Urmia has decreased from 1270.64 to 1270.38 cm in 2011, showing a 0.26 cm decline in 1 year. Some researchers believe that the dam construction on the upstream rivers is the main contributing factor in declining of the Urmia Lake water level [53,54]. At the same time, the water authorities claim that climate change is the main reason [24,55].

According to Sabzehee et al. [56] more than 22,000 deep groundwater wells have been dug in the basin of Lake Urmia for irrigation purposes and domestic consumption from 1988–2014 [22], resulting in the reduction of the lake water in this period. These deep groundwater wells are for irrigation by farmers. Apart from mismanagement of water resources, low-efficient irrigation systems are the main cause for the high water use rates in the study area. The on-farm water usage in the region is high, and irrigation has low efficiency. For surface irrigation, which is mainly used for wheat, barley, and alfalfa, the land is divided into long narrow parallel strips separated by earthen banks. In addition to losses from unlined irrigation canals, the highest losses are at the farm level from evaporation caused by inefficient irrigation practices (surface irrigation methods) and percolation into the shallow aquifer [21]. Garden and agricultural land development around the lake and decreasing trend of flow regime of rivers discharging into the lake are other important reasons. Based on their research, during the past 30 years, land cultivation around the basin has risen from 150,000 ha to 415,000 ha.

The findings of this research that explain the reasons for shrinking of Lake Urmia are supported by Hassanzadeh et al. [24] who demonstrated that climate change (65%), damming (25%), and low precipitation (10%) have contributed to the lake shrinking. In our study, we have also demonstrated that climate change and dam construction play significant roles.

**Figure 9:**
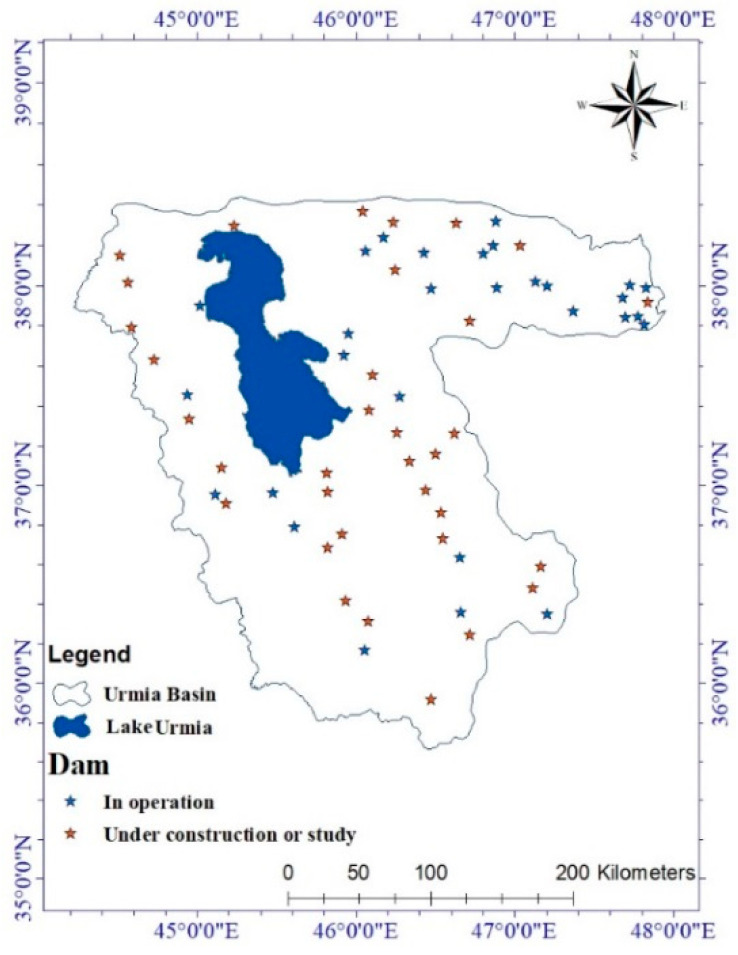
Dam locations of the Urmia Basin.

A remarkable decrease in rainfall and rivers’ water, especially in warmer months, have caused challenging problems for farmers to supply water for irrigation purposes. Therefore, damming on the main rivers to store water during rainy seasons was the only solution adopted by decision makers, which has a great role in subsiding the lake water level. Analysis of the data obtained from the IWRMC clearly shows the number of dams in operation and under construction, implying a total number of 29 active dams in the basin and 34 dams under construction (Figure 9). The latter can further cause the water level decline in the near future.

### 5.2. The Consequences of Shrinking of Lake Urmia

Due to the increased water usage in different parts of the Urmia Basin, nonobservance of sustainable development concepts, climate change, and considerable dam construction, the hydrologic balance of Lake Urmia has been completely perturbed [57]. This has led to a substantial loss of the water level in Lake Urmia. Continuous declining of the water level will trigger significant damages to the environment and could result in complete collapse of the regional ecosystem, as well as the migration of the local population. The destruction of the Lake Urmia National Park is the worst effect of shrinkage of Lake Urmia [25]. Lake Urmia is a national protected area registered in the UNESCO’s World Heritage List for being the world’s second-largest saline lake and home to diverse flora and fauna. Lake Urmia is a safe harbor for 62 species of archaebacteria and bacteria, 42 kinds of microfungi, 20 species of phytoplankton, 311 kinds of plants, five species of mollusca, 226 species of birds, 27 species of amphibians and reptiles, and 24 species of mammals [58,59,60]. The declining water level increased the concentration of saline solution from 300 to about 400 g/L [61,62]. In this case, no aquatic species can live in the lake. The lake is the habitat for many migratory birds that come from Western Europe, Siberia, and other areas in wintertime.

#### 5.2.1. Effects on Human Population

**Figure 10:**
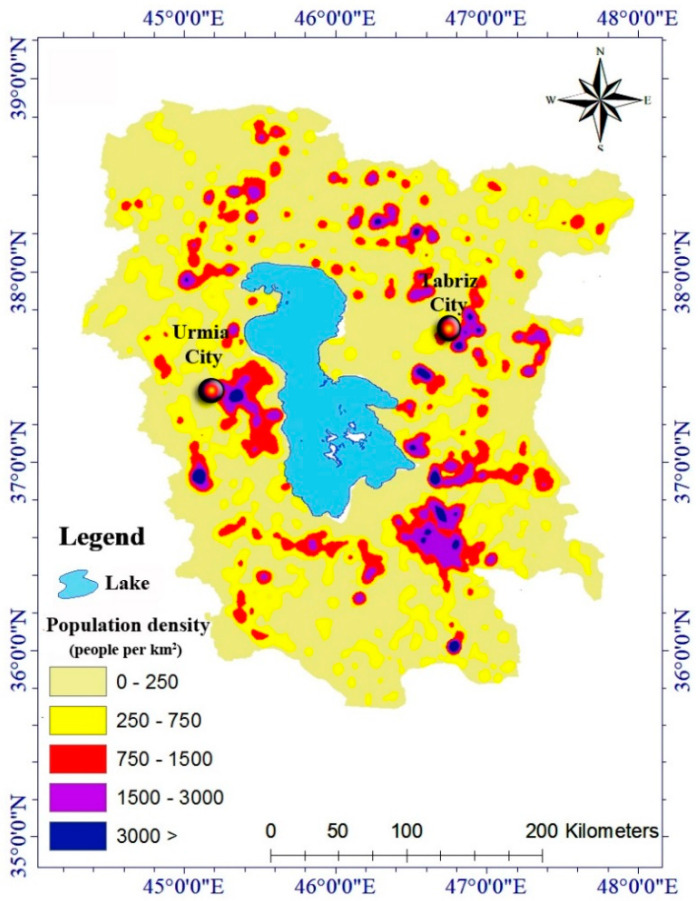
Population density (people per km^2^) around Lake Urmia.

Currently, more than 6 million people live within a buffer of less than 50 km around Lake Urmia. If the lake dries up, the entire population will be affected by salty storms. Furthermore, this phenomenon can be one of the biggest challenges in Iran and, perhaps, the world. Tabriz and Urmia are two big cities and metropolises of Iran with a population of more than 2.5 million people located less than 50 km from the lake. As lake levels decline, a saltwater desert with an area of more than 400 km^2^ will be generated [63] (Figure 10). Once the lake has dried out, it will then considerably affect the weather condition, resulting in salty winds in neighboring provinces, and will have notable impacts on human health [58].

**Figure 11:**
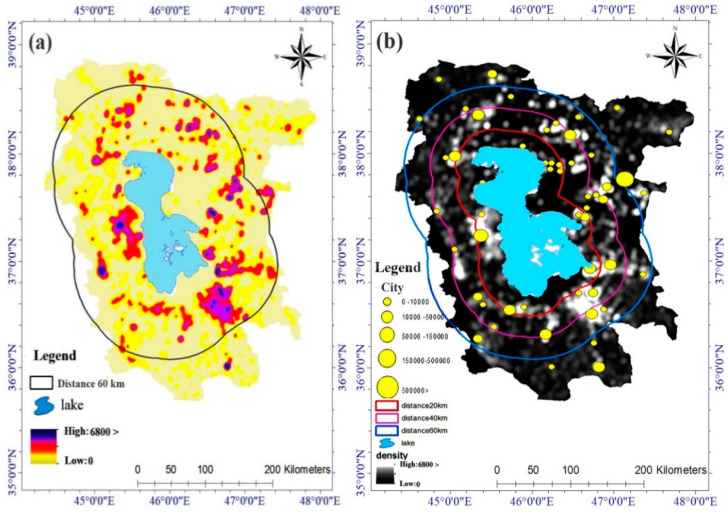
The impact of salt storms on the societies; (**a**) focal radius 60 km from Lake Urmia and (**b**) the radius of the salt storm crisis on human centers.

According to research by the Iranian Environmental Protection Agency, areas within 400 km from the lake are affected by salt storms. If we consider this distance to be one-tenth of the current distance and less than 60 km, about 5 million people will be affected by severe salt storms. Here, about 2 million people are located less than 20 km from the lake, which could be forced to migrate in the future. About 10 middle-sized cities with a population of more than 50,000 inhabitants and the Urmia metropolis, with a population of more than 800,000 inhabitants, are located less than 20 km from the lake. The metropolis of Tabriz with a population of 2 million and about 2 million inhabitants of 30 cities located less than 60 km from Lake Urmia are also affected by severe salt storms (Figure 11).

#### 5.2.2. Effects on Climatology

Zarghami et al. [21] showed that with the shrinking of the lake, major climate change could occur in the northwest of Iran in the next few decades. The average annual temperature in the northwest of Iran will reach to about 23 °C in the future and the annual precipitation will decrease by 3%, which will cause the area’s climate to change from semidesert to desert.

#### 5.2.3. Effects on the Economy

The area around Lake Urmia involves 204,000 ha of agricultural land and gardens that account for one of the main agricultural centers in Iran. Shrinking of Lake Urmia makes all these agricultural areas unproductive. Most agriculture around Lake Urmia is dependent on irrigation. The main source for agricultural water supply is surface and underground water resources [24]. In the current situation, there is a lot of exploitation of water resources and there is no balance between the amount of water withdrawn and recharged [21,64], which has caused a sharp drop in groundwater level around Lake Urmia [11,24,65]. The high rate of water extraction from the lake will cause the scarcity of water in the near future. The exploitation of water resources in the Urmia Basin is unstable, lacking proper planning and management, which will damage the highly vulnerable ecosystem of the lake, local people, as well as agriculture and livestock. Agriculture and livestock production are the only sources of income for local people. The shrinking of the lake makes agriculture and livestock farming unsustainable and people will be unemployed.

## 6. Conclusions

Lake Urmia is the world’s second-largest salty lake. Unfortunately, during the last two decades, the water level of the lake has considerably decreased in about 2745.37 km^2^ in 2017 compared to 2000. In this work, we acquired, processed, and analyzed Landsat 7 ETM^+^ satellite data for 2000, 2010, and 2017 using ENVI and ArcGIS 10.3 (ESRI, California, United States). Moreover, after trying different algorithms (i.e., Maximum Likelihood, Minimum Distance, Spectral Angle Mapper, Support Vector Machine, and Artificial Neural Network), the Maximum Likelihood model was identified as the best algorithm for classification. The overall accuracy and kappa coefficient for all classified maps were high. Decreased precipitation and increased temperature, damming, groundwater exploitation, irregular development of wells, and agricultural land development around the lake are among the most causative factors toward reduction of the water level in Lake Urmia. The declining water level will have a substantial influence on the environment and may result in a complete collapse of the regional ecosystem as well as migrating of the local human population. Moreover, the shrinking of Lake Urmia affects the weather condition and will result in salty winds in neighboring provinces, which have severe negative effects on human health. Furthermore, the destruction of Lake Urmia National Park is the worst effect of the shrinking.

Finally, this research has proposed a few possible alternatives to avoid the abovementioned crisis, including: (1) to let the flow of sufficient water from constructed dams, as the transfer of 300 million cubic meters water from Zarrineh River and Simineh River basins to Lake Urmia can change the lake’s condition towards a better status in terms of water level availability. It is highly recommended to design new irrigation management methods based on the new situation of the area since that in the Urmia Basin, the area under irrigated cultivation has increased by 19,363 ha from 1984 to 2017, which has greatly decreased the water entering into Lake Urmia, thus resulting in decrease of the water level of the lake, (2) to control more damming in the basin by optimal use of rainwater and maintenance and strengthening of vegetation and construction of check dams above the lake, and finally, (3) to avoid excessive groundwater harvesting around the lake by optimizing water resource management in agriculture and increasing the water use efficiency with a vital role for water resource conservation.

## Figures and Tables

**Table 1:** Characteristics of the images used.

Year Path & Row	2000	2010	2017
Path 168, row 34	18/10/2000	11/08/2010	01/10/2017
Path 169, row 33	22/08/2000	18/08/2010	08/10/2017
Path 169, row 34	22/08/2000	18/08/2010	08/10/2017

**Table 2:** Land area (km^2^) by different supervised classification algorithms in 2000.

Model	OA (%)	Kappa	Island	Salt Bank	Water Body
SVM	95.729	0.951	97.200	0.000	94.348
MD	93.232	0.932	94.301	0.000	91.638
SAM	88.631	0.881	87.621	0.000	89.312
NN	94.040	0.946	91.233	0.000	98.93
ML	97.918	0.963	98.231	0.000	97.436

SVM: Support Vector Machine, MD: Minimum Distance, SAM: Spectral Angle Mapper, NN: Neural Network, and ML: Maximum Likelihood.

**Table 3:** Land area (km^2^) by different supervised classification algorithms in 2010.

Model	OA (%)	Kappa	Island	Salt Bank	Water Body
SVM	95.719	0.951	93.200	95.420	98.348
MD	93.232	0.932	94.301	90.519	96.638
SAM	88.633	0.881	90.621	85.223	89.312
NN	94.047	0.946	93.233	92.852	97.93
ML	97.620	0.957	97.131	96.562	98.436

SVM: Support Vector Machine, MD: Minimum Distance, SAM: Spectral Angle Mapper, NN: Neural Network, and ML: Maximum Likelihood.

**Table 4:** Land area (km^2^) by different supervised classification algorithms in 2017.

Model	OA (%)	Kappa	Island	Salt Bank	Water Body
SVM	94.793	0.922	91.200	97.420	95.348
MD	89.320	0.912	85.801	88.913	92.838
SAM	87.631	0.801	80.621	90.223	91.312
NN	95.042	0.916	91.233	96.852	98.930
ML	96.302	0.938	96.021	97.185	97.326

SVM: Support Vector Machine, MD: Minimum Distance, SAM: Spectral Angle Mapper, NN: Neural Network, and ML: Maximum Likelihood.

**Table 5:** Accuracy assessment for the produced maps.

Accuracy Assessment	Year 2000	Year 2010	Year 2017
Kappa	0.9634	0.9574	0.9384
Overall accuracy	97.9187%	97.6198%	96.3021%
